# Mendelian-inherited heart disease: a gateway to understanding mechanisms in heart disease Update on work done at the University of Stellenbosch

**Published:** 2009-02

**Authors:** PA Brink, JC Moolman-Smook, VA Corfield

**Affiliations:** Department of Medicine, University of Stellenbosch, Stellenbosch; Department of Medical Biosciences, US/MRC Centre for Molecular and Cellular Biology, University of Stellenbosch, Stellenbosch; Department of Medical Biosciences, US/MRC Centre for Molecular and Cellular Biology, University of Stellenbosch, Stellenbosch

## Abstract

**Summary:**

The presence of founder effects in South Africa for many single-gene diseases, which include heart diseases such as progressive familial heart block types I and II, hypertrophic cardiomyopathy and the long QT syndromes, afforded us the opportunity to identify causal genes and associated mutations through genetic mapping and positional cloning. From finding the genes, the emphasis has shifted to elucidating how primary defects cause disease and recognising factors that could explain the often pronounced phenotypic variability seen in persons carrying the same inherited defect. In some of these diseases, sudden unexpected death has been a frequent occurrence in young, apparently healthy individuals who had not been aware that they had inherited an underlying risk. Herein, we review progress in identifying genes, mutations and risk factors associated with the diseases mentioned.

Nature is nowhere accustomed more openly to display her secret mysteries than in cases where she shows traces of her workings apart from the beaten path; nor is there any better way to advance the proper practice of medicine than to give our minds to the discovery of the usual law of nature by the careful investigation of cases of rarer forms of disease. For it has been found in almost all things, that what they contain of useful or of applicable nature, is hardly perceived unless we are deprived of them, or they become deranged in some way.

William Harvey, 1657

## Summary

The increasing availability, in the latter parts of the previous century, of DNA polymorphisms (markers) with known chromosomal locations allowed researchers to follow the inheritance of any chromosomal segment in families.^1^ With gene location searches no longer restricted to blood groups, the HLA system and serum protein polymorphisms, the position of any disease phenotype that segregated in a Mendelian fashion could be addressed. An early success with the new resources was with Huntington’s chorea. This autosomal dominantly (AD) inherited condition, which, besides the onset of the movement disorder, is also associated with dementia, was mapped to chromosome 4.^2^ Knowledge of the approximate chromosomal position immediately provided the means to identify at-risk and not-at-risk individuals in families, using linked DNA markers. At the same time, it paved the way to identifying the causal gene by systematically exploring genes, both known and at the time still unknown, at the identified chromosomal segment for the disease-causing mutation. 1 Appropriately named ‘positional cloning’, this approach became a paradigm for discovering the cause of many of the more than 6 000 known Mendelian-inherited characteristics, most with no known pathophysiological mechanisms.^1^

In this milieu, confronted with an AD inherited cardiac bundle branch disorder, progressive familial heart block type I (PFHBI),^3^ and with no idea how to approach the cause from a pathophysiological rationale, the logical step was positional cloning. In this way, the condition was mapped to chromosome 19q13^4^ and a novel causal gene, *TRPM4*, a member of the transient receptor potential melastatin (TRPM) family of ion channel genes, was recently identified (manuscript submitted).

Developing a focus on inherited heart disease and using molecular genetics to identify causal genes and associated mutations, we turned our attention to hypertrophic cardiomyopathy (HCM) and the long QT syndrome (LQTS), where either single individuals or families with more than one affected member were available. It was also apparent that, as these disorders are more commonly reported than PFHBI, there were better opportunities for international collaboration in this fast-evolving field. Families were expanded by cascade screening and, when necessary, ancestral ties identified by genealogical studies.

**Fig. 1.**
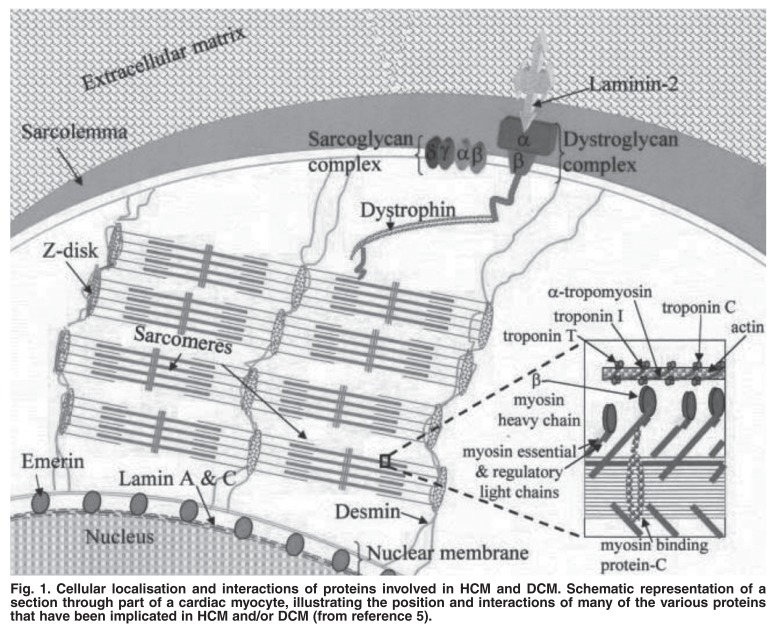
Cellular localisation and interactions of proteins involved in HCM and DCM. Schematic representation of a section through part of a cardiac myocyte, illustrating the position and interactions of many of the various proteins that have been implicated in HCM and/or DCM (from reference 5).

HCM shows an AD pattern of inheritance in a majority of cases and is characterised primarily by thickening of the ventricular wall in the absence of other hypertrophy-predisposing conditions.^5^ LQTS is recognised by episodes of syncope and a prolonged QT interval and can either be inherited in an AD manner (Romano-Ward syndrome) or autosomal recessive (AR) manner where it is associated with deafness (Jervell Lange-Nielsen syndrome).^6^ Both HCM and LQTS, together with a number of other diseases, many of them inherited in a Mendelian fashion, are important causes of sudden unexpected death (SUD) in the young, apparently well person.^7^ In the case of both HCM^8^ and LQTS,^9^,^10^ several hundred mutations, each in a number of different genes, cause the respective phenotypes. HCM-causing mutations occur in 14 causal genes, with 12 of these encoding of the cardiac sarcomere, the contractile apparatus of the heart (Fig. 1).^5^,^8^,^11^ In the case of LQTS, 12 genes have been identified, which encode proteins that form ion channel sub-units or proteins that affect ion channel functioning.^6^,^10^

In HCM, the causative genes most commonly involved are those encoding the cardiac β-myosin heavy chain (*MYH7*; thick filament), cardiac troponin T (*TNNT2*; thin filament) and cardiac myosin-binding protein C (*MYBPC3*; thick filament)^5^,^8^,^11^ (Fig. 1); disease-causing mutations have been detected in these three genes in South African families with AD patterns of inheritance.^12^-^14^ Parallel attempts to identify South African families segregating dilated cardiomyopathy (DCM) have not been successful to date (unpublished observation). In general, DCM does not behave as a single gene disease, but globally where families segregating DCM in a Mendelian inheritance pattern have been identified, the molecular cause has often been found to be related to the cytoskeleton of heart cells or to molecules that play a role in stabilisation of the nuclear membrane, cell–cell contact or connections in between (Fig. 1).^5^ These findings may yet hold importance for progressive familial heart block type II (PFHBII), an atrioventricular (AV) nodal disorder, which was described concomitantly with PFHBI^3^ and which has been mapped to chromosome 1q32.2-32.3 by the Stellenbosch group.^15^ A follow-up study found that this condition is also often associated with DCM.^16^

The phenotypic combination of AV nodal disease and DCM has often been associated with defects of the cytoskeleton. Therefore, once the disease had been mapped to chromosome 1,^15^ a compelling candidate gene in the search area was the lamin A/C encoding gene (*LMNA*), which is a cause of Emery-Dreifuss syndrome, a form of muscular dystrophy associated with DCM and conduction defects, as well as a similar isolated heart disease.^5^ However, further high-resolution mapping placed *LMNA* outside the PFHBII-containing interval,^15^ and the search for the causative gene continues.

Worldwide, the genes most commonly encountered as the cause of LQTS are *KNQ1* and *KCNH2*, which both encode potassium channels, and SCN5A, a sodium channel-encoding gene. Depending on the gene involved, which is to some extent reflected in the phenotype,^17^ the disease is sub-classified as LQT1-LQT12, for example *KCNQ1:LQT1, KCNH2:LQT2.* Mutations have been detected in South Africa in both *KCNQ1* and *KCNH2*, as well as in *KCNE1* (Durrheim, unpublished results).

The motivation for mapping and identifying disease-causing genes and associated mutations is that, not only will one be able to predict the recurrence of risk in families, but one would also be able to study and understand the diseases better. However, although identifying mutation carriers in affected families is a simple technicality, prediction has proved less tractable. Even with closely linked markers or with the mutation itself in hand, it soon became clear to us that, in all of the cardiac diseases studied, not all mutation carriers (MCs) would manifest disease by clinical criteria. In HCM, as much as 30% will have no clinical evidence of disease, yet will have increased risk of untoward events, for example SUD.^5^ Therefore, the disease allele (genotype) imparts the major risk, but is not the sole determinant of risk.

A further surprise was the extent to which some HCM and LQTS disease alleles displayed founder effects, as seen in PFHBI (Table 1). One of the reasons for a founder effect is a population bottleneck where a reduction in population numbers, e.g. caused by population decimation or by a migratory event, leads to reduction in genetic diversity and to different allele frequencies. The new expanding population will then have a genetic mix different to that of the parent population(s). Such migrations did occur with the Dutch occupation of the Cape of Good Hope in 1652 and also with the importation of slaves from Madagascar, east Africa and the Far East. Many founder effects have been described, especially in the descendants of the Dutch settlement (Dutch, French, German), the Afrikaner population.18

**Table 1:** Founder Mutations Detected In Stellenbosch Group’s Investigations

*Disease*	*Gene*	*Mutation*	*Number of families*	*Number of mutation carriers*
PFHBI	TRPM4	E7K	13	84
HCM	TNNT2	R92W	12	25
HCM	MYH7	A797T	14	80
HCM	MYH7	R403W	3	31
LQTS	KCNQ1	A341V	23	172

Diseases, genes and mutations for which strong founder effects have been identified. PFBHI = progessive familial heart block type I; HCM = hypertrphic cardiomyopathy; LQTS = long QT syndrome.

**Fig. 2.**
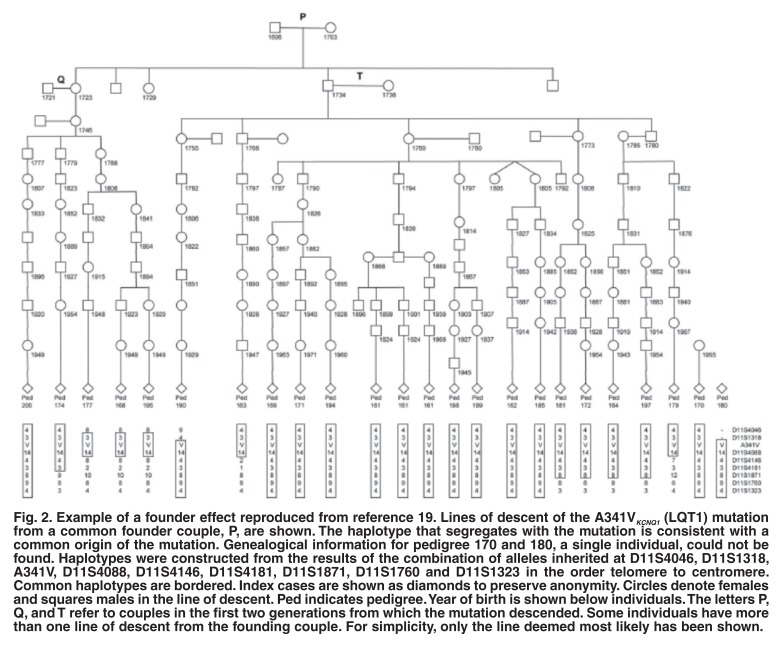
Example of a founder effect reproduced from reference 19. Lines of descent of the A341V_KCNQ1_ (LQT1) mutation from a common founder couple, P, are shown. The haplotype that segregates with the mutation is consistent with a common origin of the mutation. Genealogical information for pedigree 170 and 180, a single individual, could not be found. Haplotypes were constructed from the results of the combination of alleles inherited at D11S4046, D11S1318, A341V, D11S4088, D11S4146, D11S4181, D11S1871, D11S1760 and D11S1323 in the order telomere to centromere. Common haplotypes are bordered. Index cases are shown as diamonds to preserve anonymity. Circles denote females and squares males in the line of descent. Ped indicates pedigree. Year of birth is shown below individuals. The letters P, Q, and T refer to couples in the first two generations from which the mutation descended. Some individuals have more than one line of descent from the founding couple. For simplicity, only the line deemed most likely has been shown.

In almost all cases, a founding couple who lived prior to 1730 can be identified, e.g. in PFHBI (E7K_TRPM4b_), a couple in the eastern Cape (13 families; 84 MCs identified),^4^ and in LQT1 (A341V_KCNQ1_), a couple in the southern Cape (22 families; 172 MCs) (Fig. 2).^19^ The associated haplotype (chromosomal marker background) supports the notion that these two mutations are identical by descent (same mutation; same mutation event) for the respective diseases. In HCM, three mutations are commonly found in the HCM population, R92W_TNNT2_ (12 families; 52 MCs); A797T_MYH7_ (14 families; 80 MCs); R403W_MYH7_ (3 families; 31 MCs), again with molecular genetic evidence that they are founder effects.^13^ The A797T_MYH7_ mutation is interesting as it represents a mix of families of mixed ancestral (MA) and Afrikaner descent, while R92W_TNNT2_ and R403W_MYH7_ are only found in families of MA. Genealogical studies have not been done and may be difficult, as the history of the founding of South African sub-population groups may present challenges in ancestry tracing.

Having many individuals sharing the same disease-associated mutation offers the opportunity to observe phenotypic differences influenced by environmental or genetic factors other than the primary mutation. Furthermore, as implied at the outset of our studies, knowing the causal gene and its associated mutations creates opportunities to design laboratory-based experiments to facilitate further understanding of specific diseases.

In trying to tease out factors influencing disease expression, we generally follow a strategy of comparing selected clinical measurements of MCs with non-mutation-carrier relatives (NMCs), as genetically or environmentally driven differences between families in demographic and other characteristics which influence cardiac function and structure may exist. Where there are large enough numbers of subjects, as with the three HCMcausing mutations, we also compare MCs with different mutations with each other.

## Severity of the LQT1 A341V_KCNQ1_ mutation

**Fig. 3.**
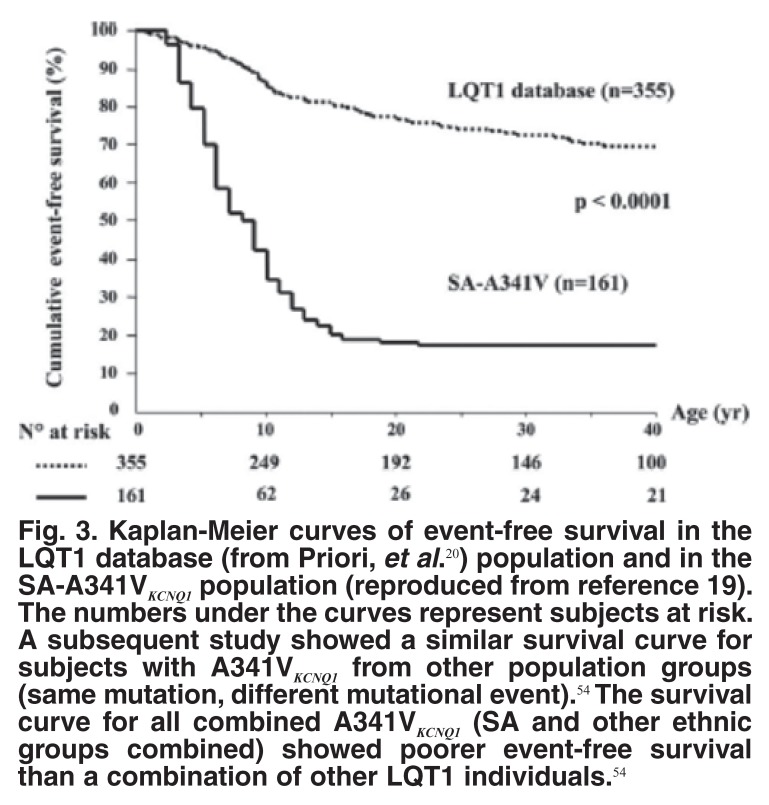
Kaplan-Meier curves of event-free survival in the LQT1 database (from Priori, et al.^20^) population and in the SA-A341V_KCNQ1_ population (reproduced from reference 19). The numbers under the curves represent subjects at risk. A subsequent study showed a similar survival curve for subjects with A341V_KCNQ1_ from other population groups (same mutation, different mutational event).^54^ The survival curve for all combined A341V_KCNQ1_ (SA and other ethnic groups combined) showed poorer event-free survival than a combination of other LQT1 individuals.^54^

The degree of clinical severity associated with A341V_KCNQ1_ was quite surprising, with 79% of MCs becoming symptomatic, versus 30% for all LQTS from an international database. Persons with the A341V mutation became symptomatic at an earlier age and experienced more LQTS-related death (Fig. 3).^19^ A partial explanation is that in patch-clamp experiments,^19^ A341V has a moderate dominant-negative effect and not a pure loss of function as previously suggested. Arrhythmic risk was even higher when the A341V carriers were compared with those harbouring *KCNQ1* mutations with a strong dominant-negative effect, suggesting an additional effect that we are not currently considering.^19^

## Influence of QTc and autonomic nervous system on disease severity in A341VKCNQ1

Virtually every A341V_KCNQ1_ MC with a QTc > 500 ms has episodes consistent with this being recognised as a major risk predictor.^20^ However, in those with a QTc < 500 ms, a number of adults have never experienced any attacks, and this was associated with lower resting heart rates in our study.^19^,^21^ Intuitively, one assumes the low heart rate to be due to increased vagus tonus. However, ‘relatively low’ BRS is significantly more present in asymptomatic MCs,^21^ contrary to the experience with post-myocardial infarction (MI) subjects where lower degrees of BRS (poor vagus responses) have been associated with a higher frequency of sudden death.^22^ This combination of lower heart rates and lower BRS signifies a blunted autonomic response. This response would, in theory, avert rapid heart rate changes which are potentially dangerous in LQT1 individuals with reduced I_Ks_ repolarising channel and inappropriate shortening of the QT interval.^21^ We have identified a physiological risk modifier with opposing effects in different arrhythmogenic substrates.^21^

## Pregnancy risk in LQT1

When comparing pregnancies in South African A341V_KCNQ1_ MCs and NMCs and in MCs on and off β-blocker therapy in ninemonth blocks before, during and after pregnancy, LQT1-affected pregnant women were at low risk for cardiac events and had no events when β-blockers were used.^23^ The perception of increased risk in the *post-partum* period evidenced in an earlier systematic study^24^ in non-genotyped LQTS could possibly be explained by the unrecognised presence of LQT2 individuals.^25^ By contrast, in our study, there was no excess risk of miscarriage and no segregation distortion, implying no selection against LQT1 MCs *in utero*. An excess of Caesarean sections in MCs was noticed. We surmised that the known tendency to bradycardia of LQT1 MCs possibly leads to misdiagnosis of foetal distress in a number of cases.^23^

## Genetic modifiers of left ventricular hypertrophy

In contrast to studies of the heart in hypertension, studies of HCM generally do not account for variables such as age, gender, body surface area, heart rate and systolic and diastolic blood pressure that may vary in conjunction with ventricle dimensions. In our studies, these variables were incorporated into a mixed-effect model, a model that also takes into account family relatedness, environment and polygenes. The hypothesis that variation at the angiotensin-converting enzyme 2 gene (*ACE2*) may modify the extent of hypertrophy was then tested in a group consisting of MCs and their NMC relatives. *ACE2* was found to be independently associated with the extent of left ventricular hypertrophy in HCM.^26^

## Disease progress in HCM – a longitudinal study

The conventional view is that HCM features most often develop from puberty to early adulthood and then stabilise.^27^,^28^ Longitudinal studies, however, of the progression of the disease in genotypically defined patients are sparse,^29^ in contrast to cross-sectional studies such as we and others have reported.^30^,^31^ Taking advantage of our database of genotyped HCM individuals, many of whom had been identified in the 1990s in a previous study, a follow-up investigation was undertaken for carriers of the R92W_TNNT2_ and A797T_MYH7_ mutations. In subjects with apparently mild disease, there was a positive correlation between age and interventricular septal thickness for both mutations, with more than a third of all MCs developing clinically recognised hypertrophy only after the age of 35 years.^32^ Furthermore, statistical modelling detected a complicated interplay between unidentified, possibly gender-associated factors and the causal mutation in cardiac function and survival. The period preceding the development of hypertrophy also coincided with the years in which most SUDs occurred, particularly in male R92W_TNNT2_ carriers.^32^

## Ventricular function in carriers of HCM-causing mutations with no hypertrophy

Although animal models and *in vitro* studies suggest distinct effects for different mutations,^33^-^36^ genotype:phenotype correlation studies in human HCM have generated inconsistent data.^37^ Human studies have typically included only individuals with fully developed disease, or combined such individuals with their sub-clinically affected MC relatives.^38^ Such studies may be confounded by differences in penetrance between mutations set against different genetic and environmental backgrounds.^39^ Furthermore, interpretation of the consequences of mutations on systolic and diastolic function may be confounded by the degree of underlying hypertrophy-associated cardiac fibrosis and disarray.^40^

Cardiac structural and functional parameters were compared between pre-hypertrophic South African MCs and NMC family members, with adjustment for appropriate covariates. The three founder mutations were associated with distinct functional effects, whereas the R92W_TNNT2_ correlated with a relative increase in systolic functional parameters, the A797_TMYH7_ correlated with a reduced diastolic function, and the R403_WMYH7_ with both reduced systolic and diastolic function.^41^ The observed early effects of the R92W_TNNT2_ mutation mechanistically fit with prolonged force-transients precipitated by increased Ca^2+^ sensitivity of the thin filament, and that of the *MYH7* mutations with local ATP depletion.

## Functional anatomy of myosin-binding protein C (MyBPC)

**Fig. 4.**
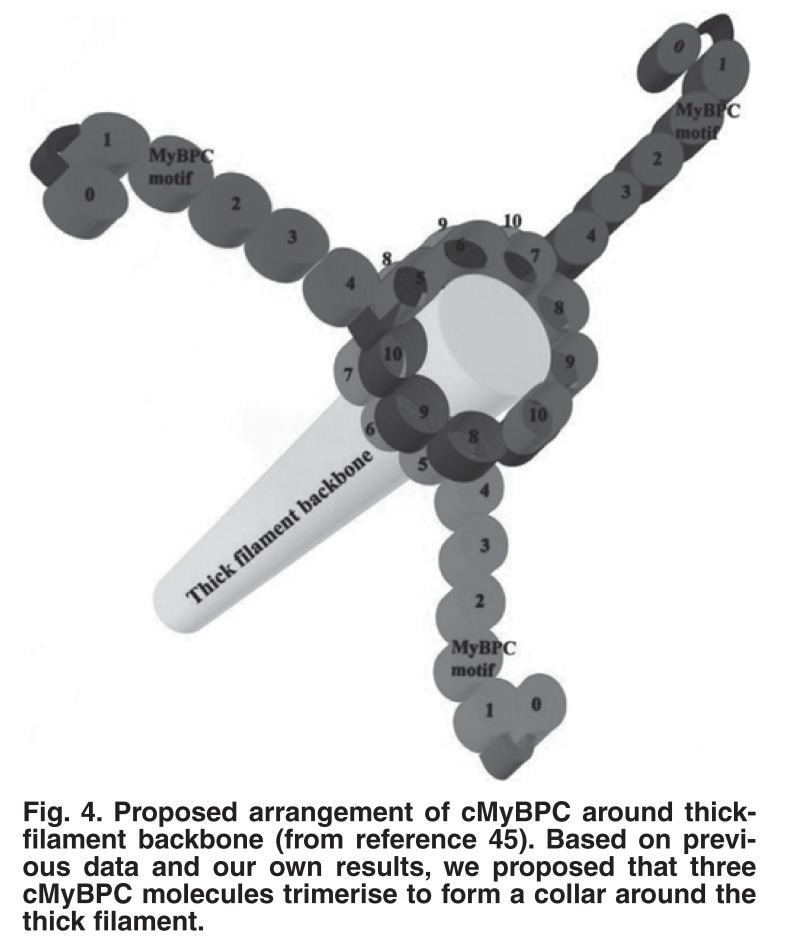
Proposed arrangement of cMyBPC around thickfilament backbone (from reference 45). Based on previous data and our own results, we proposed that three cMyBPC molecules trimerise to form a collar around the thick filament.

Myosin-binding protein C (MyBPC) was initially reported in 197342 but attracted little interest until it was associated with a human disease, namely HCM.^43^ MyBPC is a thick filament-associated protein localised to the C zones of striated muscle sarcomeres (reviewed in Flashman *et al.*^44^). A cardiac isoform, a fast-twitch and a slow-twitch skeletal muscle form exist. MyBPC contributes to thick filament structure via interactions with the light meromyosin section of the myosin rod and with titin, while also playing a role in the regulation of contraction attributed to a binding domain for the subfragment-2 portion of myosin.^45^ We performed yeast 2-hybrid experiments to identify ligands of specific MyBPC domains that were implicated in HCM pathophysiology. Important ligands detected were certain domains of MyBPC, indicating that MyBPC binds to itself. Based on previous biochemical and structural information and our data, we proposed a model where three cMyBPC molecules trimerise to form a collar around the thick filament (Fig. 4).^45^ A follow-up study clarified differences between cardiac, fast-twitch and slow-twitch muscle.^46^

## Sudden death

In the developed world, the major mode of death is sudden and unexpected, albeit even if there was pre-existing heart disease, such as a previous myocardial infarction, or the demise of elderly individuals.^47^ In the young below age 35 years, diseases other than myocardial infarction should be considered in the event of SUD.^7^,^48^ In this age group, the incidence of SUD (1:200 000) is low in absolute terms and actually falls in the same order of magnitude as death due to childhood cancer. A concern is that warning signs may be missed or misinterpreted.^49^,^50^

In our experience with LQTS in the early 1990s, in persons later diagnosed as LQTS affected, but who had a history of transient loss of consciousness (TLOC), 40% was mis-diagnosed as epilepsy and 31% was not diagnosed at all.^51^ In fact, we surmise that TLOC may even be more often misinterpreted in other population groups, as perhaps reflected in the racial composition of index cases in our LQTS, and to an extent also HCM, patient databases. To date, not a single black index case appears in the LQTS group (total 41), while persons of MA are under-represented (9.8%) when compared to national demographics for South Africa, and white subjects constitute 87.8% of the total,^52^ with other minorities making up the difference. Apart from our own, there have been no other reports of LQTS from Africa.

In our HCM database, we see a similar under-representation for black subjects – 5.1% out of a total of 176 index cases, while MA and white subjects are more equally distributed at 51.1% and 42%, and inconsistent with national demographics (total 48.6 million; black 79.2%, MA 9.0%, white 9.2%, Indian/Asian 2.6%).^53^ Genetic bias is an unlikely explanation for the demographics in our databases not following national demographics; a more likely explanation relates to missed opportunities to diagnose, missed diagnoses and misdiagnoses, and in the case of HCM, the real population drainage of our referral centre in the Western Cape.^52^

## Conclusion

Over the years, the notion has been validated that working on relatively scarce Mendelian-inherited cardiac diseases prevalent in South Africa presented an opportunity to make significant contributions in the scientific world, while benefitting many individuals and families living with the disease consequences. Furthermore, the powerful resource represented by the many South African families harbouring founder heart disease-causing mutations, and the willing co-operation of many individuals to engage in research studies has allowed us unique opportunities to gain further insights into these insidious conditions.

We have addressed the challenge that both medical professionals and the lay public are generally unaware of potential unidentified risks of SUD in the young, apparently well individual, and have played an important role in establishing PACE (Prevent Arrhythmic Cardiac Events); http://www.paceafrica.org.za/, an organisation that endeavours to increase awareness and support where necessary.
